# Effect of Notch and PARP Pathways’ Inhibition in Leukemic Cells

**DOI:** 10.3390/cells7060058

**Published:** 2018-06-14

**Authors:** Luka Horvat, Mariastefania Antica, Maja Matulić

**Affiliations:** 1 Department of Molecular Biology, Faculty of Science, University of Zagreb, Horvatovac 102A, 10000 Zagreb, Croatia; luka.horvat@biol.pmf.hr; 2 Division of Molecular Biology, Rudjer Boskovic Institute, Bijenicka 54, 10000 Zagreb, Croatia; antica@irb.hr

**Keywords:** B and T leukemia cell line, Notch, PARP1, Ikaros, Jagged, gene expression

## Abstract

Differentiation of blood cells is one of the most complex processes in the body. It is regulated by the action of transcription factors in time and space which creates a specific signaling network. In the hematopoietic signaling system, Notch is one of the main regulators of lymphocyte development. The aim of this study was to get insight into the regulation of Notch signalization and the influence of poly(ADP-ribose)polymerase (PARP) activity on this process in three leukemia cell lines obtained from B and T cells. PARP1 is an enzyme involved in posttranslational protein modification and chromatin structure changes. B and T leukemia cells were treated with Notch and PARP inhibitors, alone or in combination, for a prolonged period. The cells did not show cell proliferation arrest or apoptosis. Analysis of gene and protein expression set involved in Notch and PARP pathways revealed increase in *JAGGED1* expression after PARP1 inhibition in B cell lines and changes in Ikaros family members in both B and T cell lines after γ-secretase inhibition. These data indicate that Notch and PARP inhibition, although not inducing differentiation in leukemia cells, induce changes in signaling circuits and chromatin modelling factors.

## 1. Introduction

Today great efforts are being undertaken in the development of differentiation programs for cancer and leukemia cells to stop their proliferation and to change their biology. There are certain types of leukemia cells which can be differentiated, but still the majority of tumors are refractive to differentiation cues [1]. In this study, we investigated the possibility of cellular reprogramming by prolonged inactivation of NOTCH and PARP pathways in different leukemia cell lines of B and T origin.

Notch pathway is an important signaling pathway regulating differentiation in various tissues. Notch is a transmembrane receptor which binds ligands DELTA-LIKE and JAGGED, present on the membrane of neighboring cells. The pathway activation depends on a specific Notch receptor cleavage by γ-secretase, after ligand binding. Released Notch intracellular domain (NICD) can recruit activation complex to the promoter sequence RBPJ present in target genes. Although having only a small number of signaling elements, this pathway is modulated by numerous factors, such as expression of inhibitors, cooperation with other signaling pathways, promoters’ chromatin structure and presence of cofactors that determine the set of genes expressed in targeted cells [2,3]. In hematopoietic cells, its main role is to direct lymphocytes toward T cell line, although it also has a role in certain steps of B cell and other blood cell differentiation. Its constitutive activation was found to drive tumorigenesis in different types of leukemia, such as T cell acute lymphoblastic leukemia (T-ALL) and B cell chronic lymphocytic leukemia (B-CLL) [2,4,5].

To see whether chromatin structure and modification of signaling molecules can influence Notch signaling, in this work, we treated cells with poly(ADPribose)polymerase (PARP1) inhibitor. PARP1 is an enzyme involved in the process of polyADPribosylation, addition of polyADPribose chains on proteins and chromatin. This process is triggered by DNA damage, and its purpose is to change the chromatin structure and recruit enzymes for DNA damage repair. On the other side, more and more other functions were found to be executed by PARP1, such as transcription factors’ modification, participation in stem cell establishment and differentiation process [6]. PARP1 was found to influence Notch signaling by modifying transcriptional complex of Notch target *HES1* and downstream gene expression [7]. The interaction between HES1 and PARP1 was also found in B-ALL cells where *HES1* expression induced PARP1 activation and led to apoptosis [8]. These interactions appeared to be cell-type specific.

In this article, we describe the changes that appeared in three model hematopoietic cell lines after long-term treatment with Notch and PARP inhibitors to see whether it is possible to change the cell fate. PARP inhibition was included as potential chromatin and transcription modifier. Results show that all cell lines analyzed retained proliferation and viability. We observed an immediate decrease in expression of typical Notch target proteins in T-ALL Jurkat cells. Prolonged treatment with Notch inhibitor led to decrease in Ikaros family proteins in different leukemia cell lines, in a cell-specific way. PARP inhibition also influenced the expression of NOTCH ligands. These data indicate that Notch and PARP inhibition induce changes in signaling circuits and chromatin modelling factors regardless of typical Notch pathway activity and cell type.

## 2. Materials and Methods

### 2.1. Cell Lines and Cell Culture

Cell lines were obtained from the German Cell Culture Collection (DSMZ): Jurkat, human T cell leukemia cells, CLL, chronic lymphocytic leukemia cells and 697, human B cell precursor leukemia cells. The cells were periodically tested for the presence of mycoplasma with EZ-PCR Mycoplasma Test Kit (Biological Industry, Beit Haemek, Israel). CLL cell line was established from Epstein-Barr virus (EBV) immortalized neoplastic lymphocytes and the infection was classified as latent. Cells were cultured in RPMI-1640 medium (Sigma-Aldrich, St. Louis, MO, USA) supplemented with 10% FCS (Sigma-Aldrich, St. Louis, MO, USA), treated with Notch inhibitor DAPT (*N*-[*N*-(3,5-Difluorophenacetyl)-l-alanyl]-*S*-phenylglycine *t*-butyl ester) (Sigma-Aldrich, St. Louis, MO, USA) and PARP inhibitor PJ-34 (*N*-(6-Oxo-5,6-dihydrophenanthridin-2-yl)-(*N*,*N*-dimethylamino)acetamide hydrochloride) (Sigma-Aldrich, St. Louis, MO, USA). Control samples were treated with DMSO (Sigma-Aldrich, St. Louis, MO, USA).

### 2.2. Growth Curve and Treatment

For growth curve, cells were seeded in RPMI-1640 medium (Sigma-Aldrich, St. Louis, MO, USA) supplemented with 10% FCS at the optimum cell concentration of about 4 × 10^5^ cells/mL. Cells were counted, passaged and treated with inhibitors every other day. Cell viability was determined by counting cells under the light microscope after Trypan blue exclusion staining. The treatment cycle and downstream experiments were repeated for each cell type at least twice.

### 2.3. RNA Isolation and Preparation of cDNA

Total RNA was extracted using TRI Reagent (Sigma-Aldrich, St. Louis, MO, USA) according to the manufacturer’s instructions. Reverse transcription of isolated RNA was done using random hexamers and Primescript (TaKaRa), according to manufacturer’s protocol.

### 2.4. Quantitative Real-Time PCR

Real-time quantitative PCR (qRT-PCR) was performed using 7500 Fast Real-Time PCR System (Applied Biosystems, Waltham, MA, USA) and GoTaq qPCR Master Mix (Promega, Madison, WI, USA). All primers were designed using publicly available software’s primer3plus [9] and IDT PrimerQuest Tool [10]. The following primers were used: *HPRT* (forward: CTTTGCTGACCTGCTGGATT, reverse: TCCCCTGTTGACTGGTCATT), *HES1* (forward: GAGCACAGAAAGTCATCAAAGC, reverse: CCGCGAGCTATCTTTCTTCA), *NOTCH1* (forward: ACTCGTTCACCTGCCTGTGT, reverse: CACACCAGTGCACAAGGTTC), *NOTCH2* (forward: CTGGCAACACGCATTACT, reverse: GGCACTCATCCACTTCATAC), *JAGGED1* (forward: GACTCATCAGCCGTGTCTCA, reverse: TGGGGAACACTCACACTCAA), *PARP1* (forward: TGGAAATGCTTGACAACCTG, reverse: CATTGTGTGTGGTTGCATGA), *PARG1* (forward: TCCAGAATGGGAAAGATGTG, reverse: CTCAGCATAGCCTGTGTATTC), *IKZF1-Ikaros* (forward: CACTCCGTTGGTAAACCTC, reverse: CCTATCTTGCACAGGTCTTC), *IKZF3-Aiolos* (forward: GAAGAGCCTGAAATCCCTTAC, reverse: CCAGTATGGCTTCGCTTATG), *c-MYC* (forward: CTGCTTAGACGCTGGATTT, reverse: CTCCTCGTCGCAGTAGAAA), *EBNA2* (forward: TTCCACCTATGCCATTACCC, reverse: GCCTTGAGTCTTAGAGGGTT).

Expression of *HPRT* gene was used as an endogenous control for normalization. Efficacy of PCR reaction was calculated from the slope of the amplification curve in the exponential phase, by using linear regression software (LinRegPCR 2014.x) and was higher than 90%. Product specificity was determined by amplicon melting curve. All significant changes were confirmed on two or three biological replicas. Results were presented as fold change value [11].

### 2.5. Western Blot

Total cell extracts were prepared using lysis buffer containing a cocktail of protease inhibitors (Carl Roth, Karlsruhe, Germany), as described previously [12]. Proteins were analyzed by Western blot using chemiluminescence detection method [12]. Primary antibodies were used for detection of β actin, JAGGED1, PARP1, IKZF3 (all from Santa Cruz, Dallas, TX, USA), HES1, IKZF1, NOTCH1 and NOTCH1 cleaved (all from Cell Signaling Technology, Danvers, MA, USA). Densitometric analysis was performed using ImageJ program (NIH, Bethesda, MD, USA).

### 2.6. Statistical Methods

Data were statistically analyzed using the software package Microsoft Office. A parametric test was used for comparison of results between control and treated cells. The significance of independent two-tailed Student’s *t*-test was set at *p*-value < 0.05.

## 3. Results

### 3.1. Effect of Notch and PARP inhibition on Jurkat, CLL and 697 Cells’ Proliferation

Notch pathway is considered to be one of the main pathways directing cells toward T and not B lineage and is often deregulated in T cell leukemia. Certain types of leukemia can even be “addicted” on Notch activity and its abrogation could influence cell proliferation [13]. On the other side, a direct role of PARP1 in differentiation is still under investigation, but it is known that it can influence the structure of cell chromatin and modulate transcription factor activity [6,14]. 

**Figure 1:**
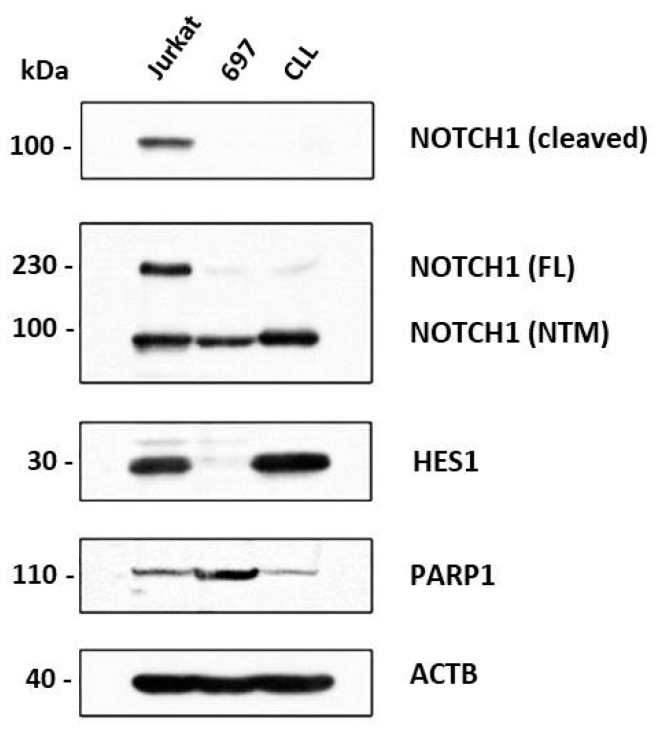
Characterization of the Notch signaling pathway in leukemic cells. Notch signaling activity in T cell line (Jurkat), B chronic lymphocytic cell line (CLL) and B cell precursor leukemia cell line (697) was analyzed by Western blot. FL: full-length; NTM: transmembrane/intracellular region; ACTB: β-actin.

Analysis of Notch signaling in model cell lines showed that this pathway was active in Jurkat T cells, as well as in B chronic lymphocytic cell line CLL, while in pre B cells 697 was not. These data could be concluded from the presence of cleaved Notch domain NICD and expression of Notch downstream target HES1 (Figure 1). 

**Figure 2:**
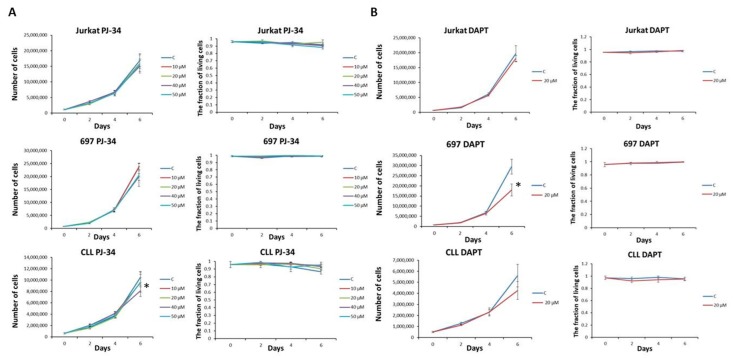
Influence of PJ-34 and DAPT inhibitors on proliferation and viability of leukemic cell lines. Jurkat, 697 and CLL cell lines were treated with PARP1 inhibitor PJ-34 (**A**) and Notch inhibitor DAPT (**B**) every second day, for six days. Total cell number and the fraction of living cells were obtained by counting cells stained with Trypan blue dye under the light microscope. The standard deviation of the four different counts is displayed. C: control cells; 10 µM, 20 µM, 40 µM, and 50 µM: concentrations of the treating agent; * *p*-value < 0.05.

Next, we analyzed cell proliferation and cytotoxicity in cells treated with different concentrations of PJ-34, the inhibitor of PARP activity [15]. We detected no cell proliferation arrest and no significant cytotoxicity in any of three cell lines, except B CLL line showing decreased proliferation after six days of treatment with 40 µM PJ-34. In parallel, treatment with 20 µM DAPT [16], γ-secretase inhibitor, did not influence cell proliferation in Jurkat cells. Proliferation of 697 and CLL cells started to decrease after the fourth day, and was significantly decreased in 697 cells after six days of treatment (Figure 2). Viability of all the cell lines was on the control level.

### 3.2. Effect of Notch and PARP Inhibition on Jurkat, CLL and 697 Cells’ mRNA Expression

**Figure 3:**
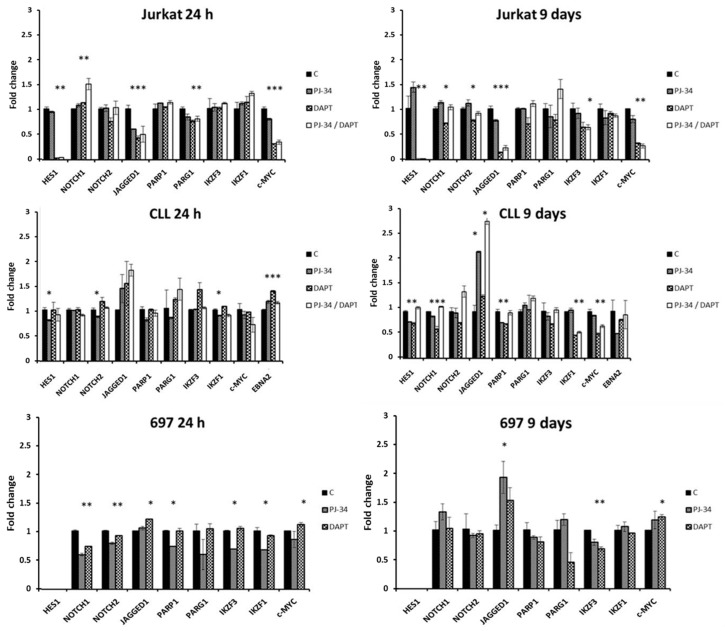
Influence of Notch and PARP pathways’ inhibition in Jurkat, CLL, and 697 cell lines. Cells were treated with PARP inhibitor PJ-34 and/or Notch inhibitor DAPT every other day through nine days, when RNA was isolated and, after reverse transcription, qRT-PCR was performed. Representative results are shown. Relative expression is presented as a fold change in comparison with untreated control sample values. As endogenous control gene *HPRT* expression was used. C: control cells; PJ-34: cells treated with PJ-34 (10 µM for CLL and Jurkat cells and 40 µM for 697); DAPT: 20 µM DAPT; PJ-34/DAPT: cells treated with combination of 10 µM PJ-34 and 20 µM DAPT; * *p*-value < 0.05.

The expression of a series of genes involved in Notch and PARP pathways was analyzed in Jurkat, CLL, and 697 cells after one and nine days of treatment with Notch and PARP inhibitors, alone or in combination, by qRT-PCR (Figure 3). 

Jurkat cells’ analysis revealed typical decrease of *HES1* expression, as being direct Notch target, in samples treated for 24 h and nine days with Notch inhibitor. Expression of *NOTCH1* and *NOTCH2* receptors showed oscillations in dependence on DAPT treatment after 24 h and nine days. *c-MYC* decreased its expression even after 24 h, and stayed downregulated for nine days of treatment with Notch inhibitor. Cells treated with DAPT had also decreased expression of *JAGGED1*, after one and nine days. PARP inhibition similarly affected its expression. *PARP1* and *PARG*, coding for an enzyme involved in degradation of polyADPribose chains produced by PARP1, had control level expression, after both types of treatment for nine days. We also tested expression of *IKZF3* and *IKZF1*, two members of Ikaros transcription factor family, which can interfere with NICD complex binding to RBPJ sequence [17]. They did not show any significant changes in expression 24 h after treatment, but after nine days of treatment with DAPT *IKZF3* expression decreased by ~40%. 

CLL cells exprimed Notch pathway molecules, receptors *NOTCH1* and *NOTCH2* and ligand *JAGGED1*, as well as HES1, a downstream Notch target, indicating unusual activity of the pathway. Inhibition of γ secretase for nine days induced only a small decrease in *HES1* and *NOTCH1* expression. Another downstream target, *c-MYC*, did not show significant differences in its expression after 24-h treatment, but after nine-day treatment with DAPT, *c-MYC* expression was decreased to ~50% of control values. While *IKZF3* expression did not significantly changed, nine days of γ-secretase inhibition induced ~50% decrease in *IKZF1* expression. Although it is known that chronic lymphocytic leukemia cells could have active Notch pathway due to Notch mutation, the inability of *HES1* to be inactivated by DAPT led us to analyze the expression of EBV protein EBNA2. Namely, B chronic leukemia cell lines are usually produced by EBV immortalization, and EBNA2 can also bind to Notch targeting sequences [18]. As we found *EBNA2* expression, we concluded that CLL cells have expression of downstream Notch target genes caused by EBNA2 binding. Although 24-h treatment caused fluctuations in *EBNA2* expression, no significant changes were observed after prolonged treatment with inhibitors. PARP inhibition increased expression of *JAGGED1*, Notch ligand by more than two times, when tested in the samples treated for nine days. Delta-like ligand 1 (*DLL1*), another Notch ligand, did not show detectable expression by PCR (data not shown). 

We also analyzed 697 cell line, originating form B acute lymphocytic leukemia and showing pre-B phenotype. These cells did not have active Notch pathway, but, as we observed indirect effects of γ-secretase inhibition in other cell lines, we treated these cells with PJ and DAPT alone. Expression of genes involved in Notch and PARP pathways was analyzed, after one and nine days of treatment. *HES1*, as Notch downstream target, was not expressed in these cells, and *c-MYC* expression was not dependent on its signaling. γ-secretase inhibition decreased *IKZF3* expression after nine days of treatment. PARP inhibition decreased expression of *NOTCH1*, *NOTCH2*, *PARP1*, *IKZF3* and *IKZF1* after 24 h, but by prolonged treatment most of these effects were lost. PJ-34 also increased expression of *JAGGED1* for nearly two times after nine days of treatment.

### 3.3. Effect of Notch and PARP Inhibition on Jurkat, CLL and 697 Cells’ Protein Expression

Proteins expressed from genes influenced by PARP and Notch inhibitors were analyzed by Western blot. Active NOTCH1 pathway and presence of NICD1 were detected in Jurkat cells, absence of NICD1 and HES1 in 697, and expression of HES1, but absence of NICD in CLL (Figure 1). 

Analysis of Jurkat cells treated for nine days with Notch inhibitor showed abrogation of HES1 expression, IKZF1 expression was equal in control and treated cells and IKZF3 was moderately decreased after treatment. In addition, following mRNA inhibition, expression of JAGGED1 on protein level was decreased. PARP inhibition did not significantly change the expression of HES1, IKZF3, IKZF1, and PARP1, and we did not observe decrease in JAGGED1 expression.

**Figure 4:**
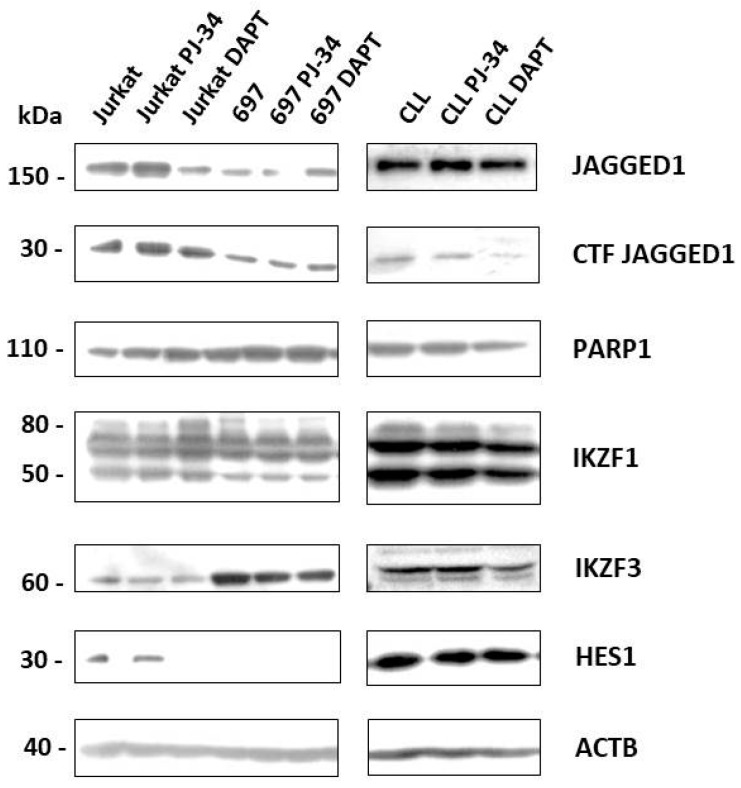
Influence of Notch and PARP pathways’ inhibition on protein expression. T cell line (Jurkat), B chronic lymphocytic cell line (CLL) and B cell precursor leukemia cell line (697) were treated with Notch pathway inhibitor DAPT (20 µM) or PARP inhibitor PJ-34 (10 µM) for nine days, when proteins were isolated and analyzed by Western blot. CTF: C-terminal fragment; ACTB: β-actin.

In CLL cells, HES1 did not change its expression after nine days of PARP and Notch inhibition. IKZF1 and IKZF3 levels were lower in DAPT treated cells. PARP expression was similar in untreated and treated cells and JAGGED1 expression was slightly increased in cells treated with PARP inhibitor (Figure 4).

In 697 cells, HES1 was not exprimed, as expected; IKZF1 was on control level; and IKZF3 showed decrease in expression after treatment with both, PARP and Notch inhibitors. We could not detect JAGGED1 increase on the protein level.

## 4. Discussion

In our experiments, we analyzed changes in a set of genes/proteins involved in PARP and Notch signaling pathways after long-term treatment of three leukemia cell lines with PARP and Notch inhibitors. We analyzed T cell line Jurkat, known for active Notch pathway, a pre B leukemia cell line without active Notch pathway, and a chronic lymphocytic cell line obtained by EBV immortalization of pre B leukemia cells. The CLL line did not have active NOTCH1 intracellular domain, but showed activity of downstream Notch target genes. Jurkat cells had a NOTCH1 mutation enabling its constant activation [19]. 

Analyzing changes in expression in cells treated with γ-secretase inhibitor DAPT, we observed inhibition of genes known to be Notch downstream targets in cells where typical active Notch pathway was present in untreated cells. We could also expect consequences of inhibition of a series of molecules which are direct substrates of γ-secretase (around 90 proteins, if expressed). Beside Notch receptors, these are Delta and Jagged ligands and amyloid precursor protein (e.g., [20]). However, even if their conformation allows γ-secretase to cut them, it is difficult to predict the effect on the gene expression as the target genes are regulated through multiple positive and negative feed-backs and promoter chromatin structure. 

Jurkat cells present typical cells with active Notch signaling, inhibited by γ-secretase. Already 24 h after pathway inhibition there was a downregulation of Notch target genes such as *HES1* and *c-MYC*. We also observed inhibition of *JAGGED1*, Notch ligand, and possible γ-secretase substrate. 

Inhibition of Notch signaling did not cause cell arrest and apoptosis and the cells remained viable. Jurkat cells belong to T-ALL resistant to Notch signaling inhibitors due to PTEN-null genotype. Namely, PTEN expression in nonmutated cells inhibits survival pathways going through Akt and PI3K and stops proliferation. Furthermore, its promoter is by feed-back loop regulated by HES1, a downstream Notch target. In Jurkat and some other T-ALL cells, this pathway is abrogated by PTEN deficiency and these cells become dependent on Akt survival pathway [21,22]. 

Another gene downregulated by Notch inhibition is c-*MYC*. This gene can also be regulated by several pathways, such as those Akt related. *c-MYC* was in CLL and Jurkat cells decreased for 50%. We could suppose that in CLL c-MYC decrease can be connected with the slowing down of cell proliferation, but in Jurkat cells proliferation was independent of c-MYC expression. Having other signaling pathways to force proliferation, *c-MYC* downregulation in proliferating cells is not uncommon in lymphocyte biology; i.e., similar features are present in some parts of germinative center of lymph nodes [23]. In addition, Notch and c-MYC could also be connected through regulatory loops involving microRNA (mostly microRNA 30a) in B and T leukemia [24]. In pre B leukemic cells, IKZF1 and IKZF3 were also found to inhibit proliferation through the decrease of c-MYC expression [25]. 

JAGGED1 is a Notch ligand whose role in the regulation of Notch pathways is still under investigation. It was found to increase Notch signaling in lymphomas, through the NOTCH2-HEY1 pathway [26]. In certain tissues reciprocal regulation of Notch pathways was found to be exerted by DLL1 and JAGGED1 [27]. Recently, it was discovered that JAGGED1 can be cleaved by the same molecules as NOTCH receptor and that intracellular domain can cause signal transduction in the host cell, competing with Notch [20]. Thus, being a substrate of γ-secretase, it is possible that DAPT inhibited its expression in Jurkat cells by feedback signaling.

Prolonged treatment with γ-secretase inhibitor caused changes in expression of not only genes which were immediately downregulated, but also those of IKZF1 family of transcription factors: in Jurkat and 697 cells IKZF3 was decreased, and in CLL IKZF1, regardless of the Notch pathway inhibition. Their role in cell regulation is still widely unknown. It is known that IKZF1 is important for directing lymphocytes toward T cell line and that its deleted forms could be found in a set of T-ALL [28]. Ikaros family proteins act as transcription factors and could have a role in a chromatin remodeling interacting with NuRD complex [29,30]. IKZF1 targets elements similar to RBPJ sequence, the downstream target of Notch signaling. On its binding sites there is often competition or cooperation between different transcription factors. It was found that IKZF1 influences the repertoire of Notch target genes in T cells targeting to RBPJ regulatory sequences [31,32] and Notch signaling loops antagonize *IKZF1* expression in T-ALL. *IKZF1* expression was found to be reduced in T-ALL patient samples with activating Notch pathway mutations. The supposed mechanism is competition for direct target promoter binding, such as on *MYC* gene [33]. IKZF1 and IKZF3 are considered tumor suppressors and their inability to perform their functions are connected with tumor development. Knockdown of Ikaros reduced B cell differentiation to plasma cells [34]. Recently a new mechanism was found by which Ikaros takes part in transcription complex with IRF4 and in this way regulates genes which should be downregulated during plasma cell differentiation [34,35]. IKFZ1 was also found to repress expression of PI3K pathway genes. These data indicate the role of this family in intricate signaling loops regulating blood cell differentiation. Possibly, inhibition of a signaling pathway stimulating proliferation needs to be balanced by inhibition of a tumor suppressor, through their signaling circuits, to retain viability and proliferation. In addition, as loss of IKFZ1 leads to ineffective tyrosine kinase inhibitor therapy in certain types of leukemia, the ability of chemotherapeutic to decrease its expression could have an influence on the final output of anticancer therapy [36].

Changes in Ikaros family members became significant after the prolonged period of γ-secretase inhibition, indicating indirect and secondary signaling. *IKZF1* promoter is extremely complex, having several distinct modules/enhancers forming regulatory units to upregulate its expression in different hematopoietic cells or during cell differentiation. These regulatory regions depend on a number of transcription factors, such as IKZF1, ETS1, c-MYC, RUNX1, etc., and several of them could be involved in positive and negative signaling loops of Notch signaling [37]. In addition, IKZF1 was also found to control IKZF3 expression [38]. 

Considering B cell lines, Notch activation can lead to pre B leukemia cells’ apoptosis [8]. On the other side, a fraction of B-CLL types can have constitutively active Notch pathway, mainly because of PEST-domain mutations. It was found that it is crucial for their survival and its abrogation could lead these cells to apoptosis [39]. Our model CLL line had active Notch signaling but did not show apoptosis after treatment with Notch inhibitor and only 20% decrease in growth was present after long-term treatment. Protein analysis showed the absence of NOTCH1 intracellular domain, NICD1, but presence of HES1 expression. We found expression of *EBNA2*, early EBV protein involved in latency type II and III, being expressed as a consequence of immortalization process. EBNA2 binds to RBPJ sequences in the genome, competing out Notch intracellular domain [18]. CLL cells showed resistance to γ-secretase inhibitor acting on upstream levels of Notch signaling. HES1 and c-MYC expression could therefore be a consequence of EBNA2 binding. EBNA2 levels did not change significantly after DAPT treatment. 

PARP1 is a multitasking protein involved in a cellular response on DNA damage as well as in transcription and differentiation processes. It can modulate the activity of several transcription factors, either through protein–protein interactions or through parpylation, addition of polyADP-ribose. Being an inhibitor of PARP activation, PJ-34 can influence only a set of PARP activities in the cell. PARP was found to be connected with Notch signaling through modification of a complex in which transcription factor HES1 takes part [7]. Kannan et al. [8] found that Notch signaling in B cell ALL, which led to growth arrest and apoptosis was regulated by HES1 modulated by PARP1. As *HES1* was not exprimed in 697 cells, PARP inhibition did not significantly influence survival and proliferation of these cells.

In T-ALL and CLL cells, the most prominent effect of PARP1 inhibition was the influence on *JAGGED1* expression. While it was upregulated in both B cell lines, in Jurkat cells, *JAGGED1* expression was decreased. One possibility is that PARP1 modulates both, activation and repression complexes on the gene promoter, in dependence on the intracellular milieu [7] and the other that PARP1 modulates different transcription factors present in different cell types, such as NFAT1, NF-κB or others [6,40,41]. PARP1 targets are also SMAD transcription factors, influencing the duration of TGF β signaling, and JAGGED1 is a known TGF β target [42,43]. On the other side, analysis of TGF β expression after PARP inhibition revealed downregulation in Jurkat cells after prolonged treatment. In other cell lines, TGF β expression was not significantly influenced by treatment (data not shown).

Finally, we could conclude that each type of leukemia and leukemia cell line has a unique net of signaling pathways connected with feedback loops and does not respond to inhibition of certain pathway in the same way. Jurkat and CLL cells, although having active downstream Notch signaling, do not respond to Notch signaling inhibitor by cell arrest or apoptosis, although inhibition of upstream signaling was present. In similar experiments with different types of tumors, certain differentiation cues did even cause changes in DNA methylation pattern and binding patterns of transcription factors, but cells re-entered the proliferation cycle when the growth factor was removed. Persistent oncogene expression made cells resistant to differentiation cues [44]. Although we showed that long-term treatment could cause changes in expression of signaling molecules, possibly other pro-survival signaling circuits dominated over Notch pathway, enabling these cells to be resistant to apoptosis and differentiation. 

Our results showed that effects of Notch and PARP inhibition, although influencing similar groups of genes in different cell lines, also greatly depend on the intracellular milieu of each cell line. Therefore, it would be interesting to analyze primary B and T cells, as well as primary leukemia cells. Similar experiments have been already done in certain subgroups of these cells. Different Notch inhibitors were shown to influence early T cell development, as well as decrease cytokine production in activated T cells [45]. Considering B cells, Notch inhibition did not influence significantly viability of normal peripheral B lymphocytes but caused depletion in marginal B zone cells in vivo [46]. In primary leukemic cells, Notch inhibition was cytotoxic for a subgroup of Notch-mutated B and T leukemic cells [39,47]. Effects of PARP inhibition were analyzed in trials testing olaparib, a PARP inhibitor used for cancer treatment. PARP inhibition on immortalized B lymphocytes did not show decrease in cell viability. At the molecular level, Martin et al. found that olaparib caused changes in expression of genes involved in stress response, protein ubiquitination and cell signaling, possibly mediated through regulation of epigenetic regulator EZH2 and NFAT signaling [48]. These processes were involved in cell differentiation and cytokine expression. Considering lymphoid tumor cells, PARP inhibition was shown to be cytotoxic for ATM-deficient cells, in vivo and in vitro [49]. All these data indicate the necessity of getting global insight into the cellular circuits governing mechanisms involving Notch and PARP in different cell types. 

## 5. Conclusions

Long-term inhibition of Notch signaling and PARP activity in three leukemic cell lines of B and T origin did not cause proliferation arrest and apoptosis, but induced changes in expression in a set of genes. Inhibition of PARP1 caused *JAGGED1* expression to change in all three types of observed lines. The γ-secretase inhibitor influenced the expression of transcription factors from the Ikaros family regardless of the Notch signaling activity, but depending on the differentiation type of lymphocyte. Further investigations are needed to explain the mechanisms of these processes, which could have an influence on chromatin structure and change the cell response on other chemotherapeutics.
